# Supplementary material for: Regulation of heterotopic ossification by monocytes in a mouse model of aberrant wound healing
Source: Nat Commun. 2020 Feb 5;11:722. doi: 10.1038/s41467-019-14172-4 (PMC7002453; doi:10.1038/s41467-019-14172-4)
Supplement: Supplementary file 1 — Supplementary Information [file 41467_2019_14172_MOESM1_ESM.pdf]

Supplemental Information:

Regulation of heterotopic ossification through local inflammatory monocytes in a mouse model of aberrant wound healing

Sorkin et al.

Supplemental Figure 1. Plasma cyto/chemokine levels. Data are shown as the median and interquartile range. Changes in cytokines and chemokines across day 3 and day 7 vs day 0 were analyzed by an analysis of variance (ANOVA) with post-hoc Dunnett test (n=3 mice/time point). Non-heteroscedastic data identified by Levene's test for homogeneity of variances were alternatively analyzed by Welch statistic and post-hoc Dunnett T3. Degrees of freedom (df or df1) across samples = 2. F statistic and significant post-hoc p-values respectively: CXCL1: 0.587, CXCL2: 0.388, CCL2: 1.259, CCL3: 2.295, CCL4: 0.178, G-CSF: 2.736, GM-CSF: 1.099, IL-1b: 6.732, p(D0 vs. D3)=0.032, p(D0 vs. D7)=0.036, IL-6: 11.394, p(D0 vs. D3)=0.009,TNF-α: 0.303, TGF-β1: 2.746, p(D0 vs. D3)=0.009,TGF-β2: 12.294, p(D0 vs. D3)=0.027, p(D0 vs. D7)=0.007, TGF-β3: 0.303, LIF: .994. *p<.05 **p<.01. Source data are provided as a Source Data file.

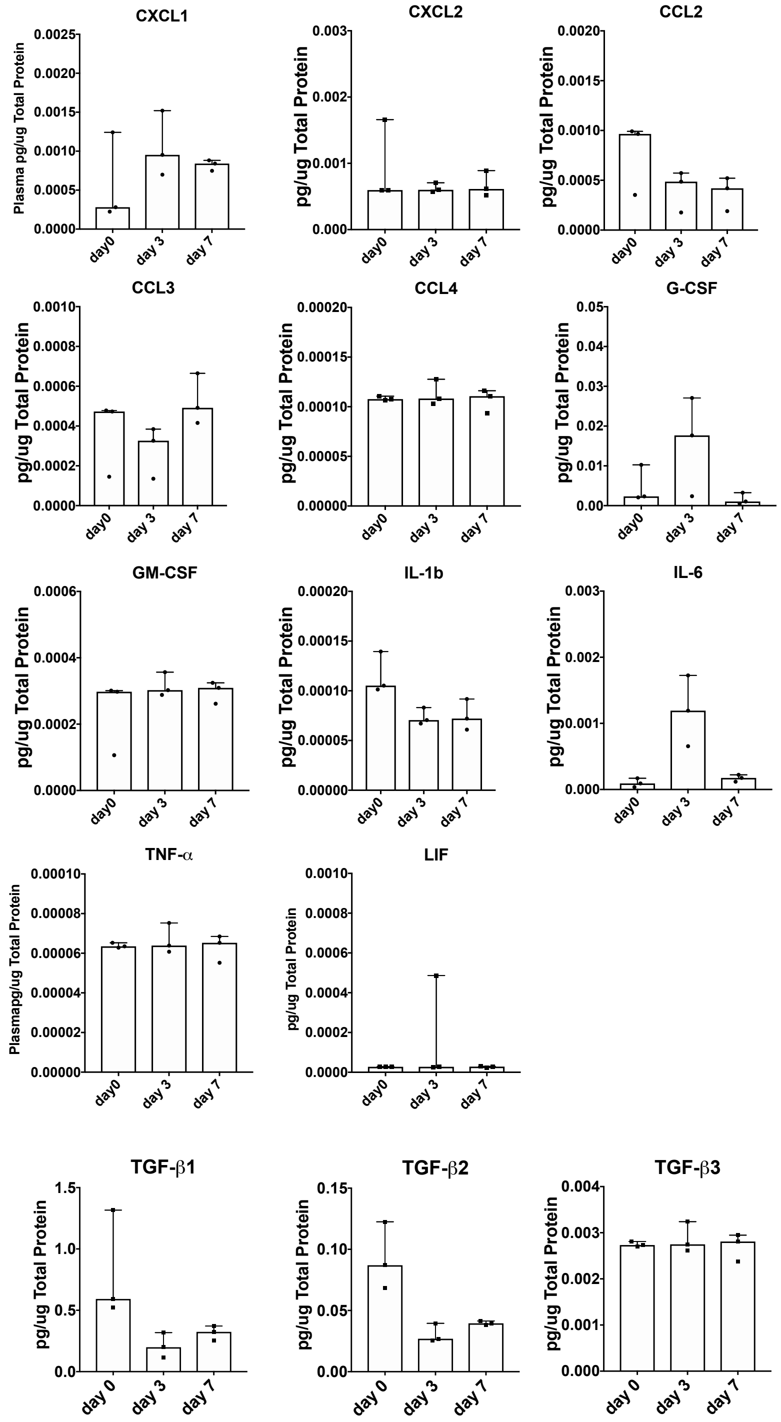

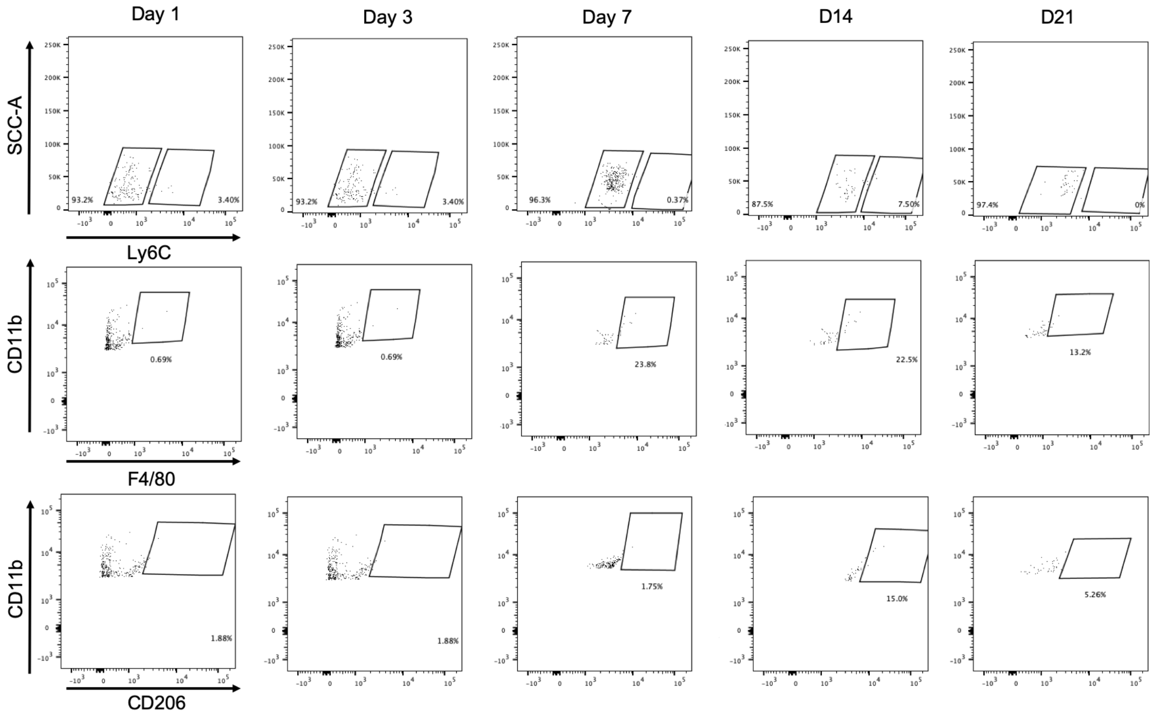

Supplemental Figure 2. Negative gates of HO site flow cytometry. Flow cytometry plots showing negative gating strategy for Ly6C, F4/80 and CD206 across the individual time points.

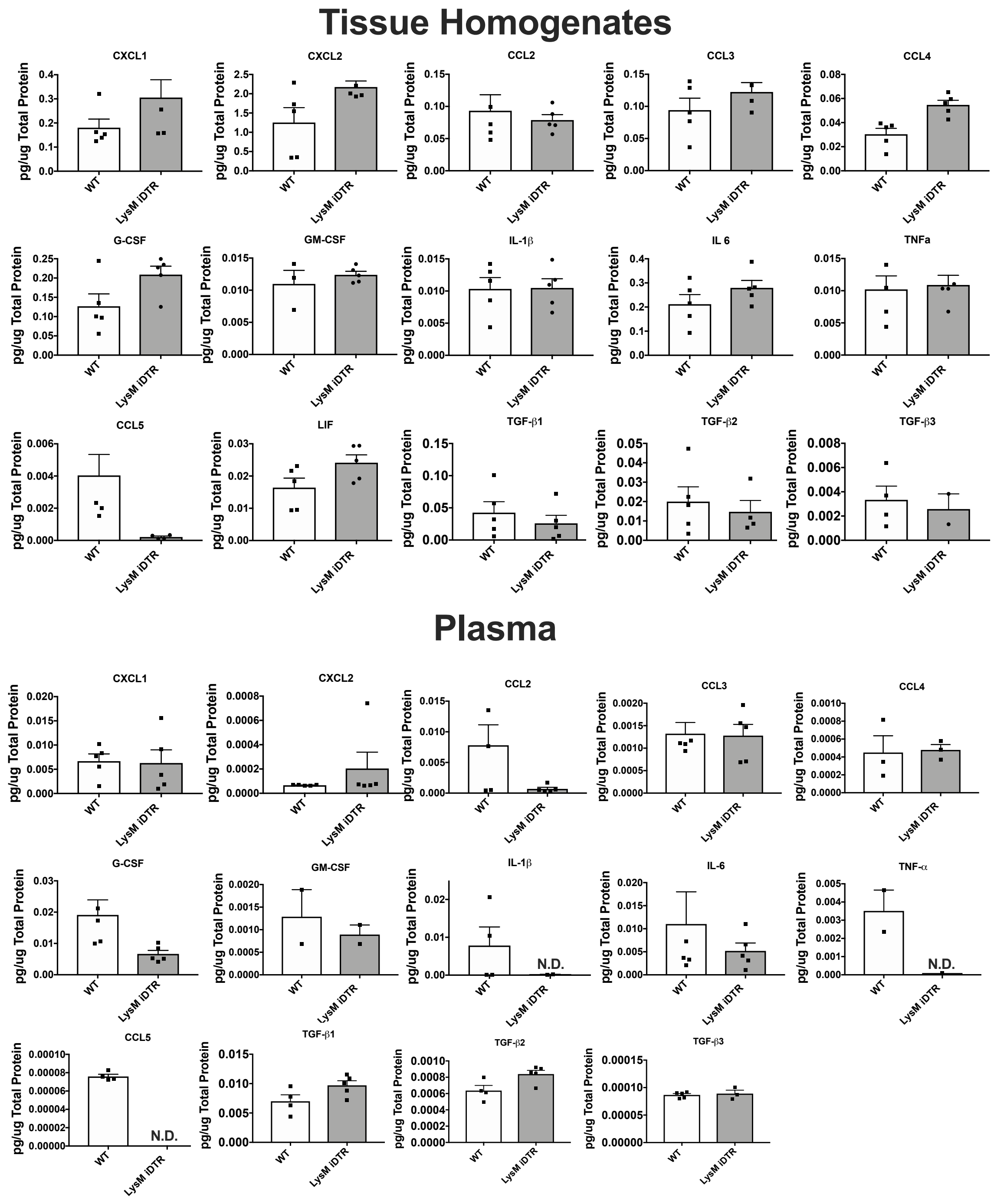

Supplemental Figure 3. Homogenate and Plasma levels of cytokines/chemokines after monocyte depletion using LysMCre-*iDTR* or control WT mice (n=5 mice/group). Injury site homogenate and plasma at day 3 from LysMCre-iDTR and liter mate control mice, where monocytes/macrophages were depleted by pre-injection of diptheria toxin (DT) two days before the B/T, the day of B/T and at day 2 after the B/T. Source data are provided as a Source Data file.

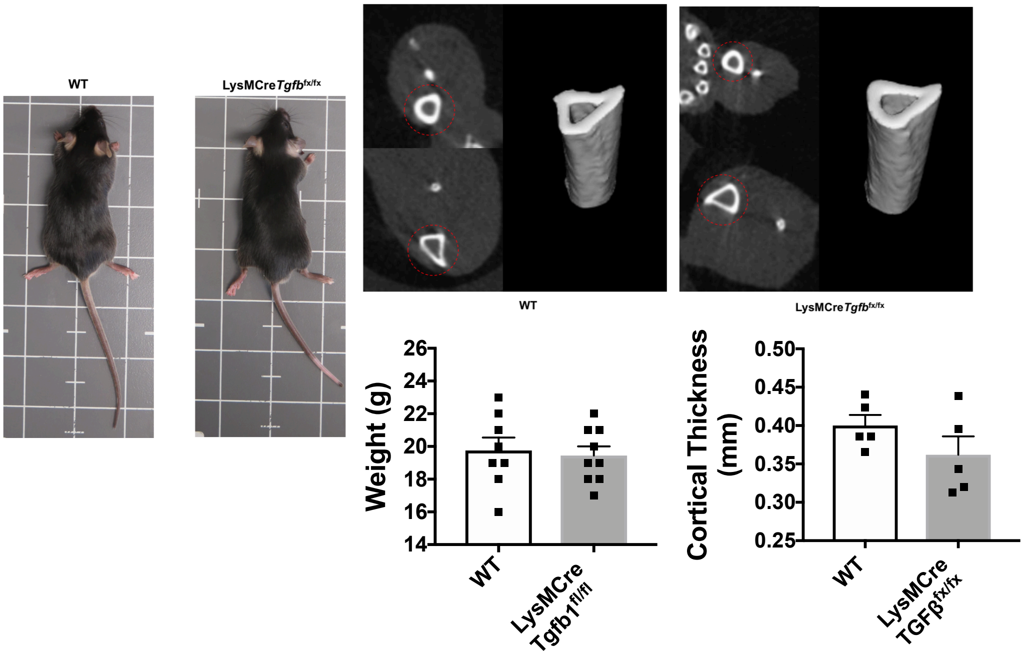

Supplemental Figure 4. LysMcre/Tgfb1$^{fl/fl}$ and wild type mice do not differ in size, weight, or tibial thickness. A. Top: images of wild type and LysMcre/Tgfb1$^{fl/fl}$ mice. Bottom: mean weights from mice in either WT or LysMcre/Tgfb1$^{fl/fl}$ groups. B. Quantification of mean cortical tibial thickness from MicroCT scans of WT and LysMcre/Tgfb1$^{fl/fl}$ mice (n=5/group, t=-0.121, df=8, p=0.907) Source data are provided as a Source Data file.

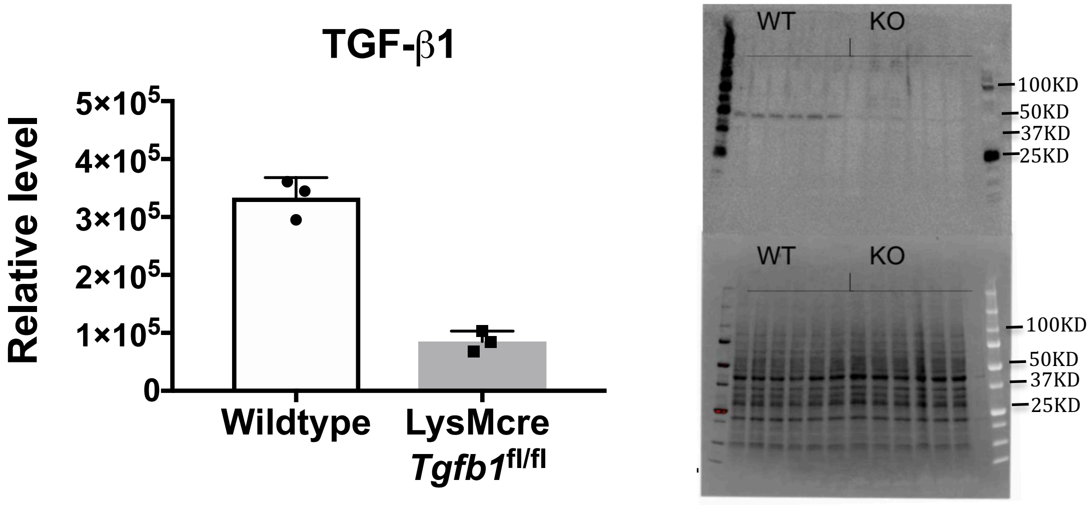

Supplemental Figure 5. TGF-β1 expression is reduced in LysMCre-*Tgfb1*$^{fl/fl}$ bone marrow derived macrophages. Bone marrow was flushed from 4-week-old WT (C57B6) or KO (LysMCre-*Tgfb1*$^{fl/fl}$) mice and macrophages induced in culture with 30ng/ml M-CSF for 5 days. Western blot revealed a significant decrease in TGF-β1. Graph represents TGF-β1 expression (seen in Western blot; right, top panel) normalized to loading signal (right, bottom panel). n=3/gp (run in duplicate; t test: p=0.0004, t=11.15, df=4) error bars represent SD. Source data are provided as a Source Data file.

Tissue Homogenate

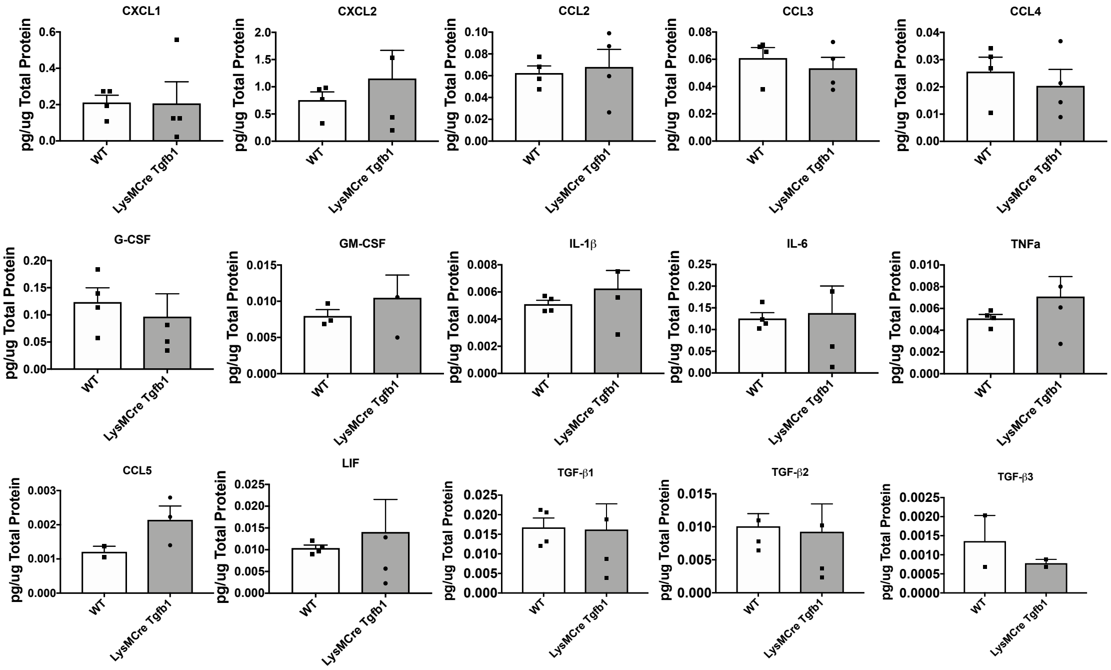

Plasma

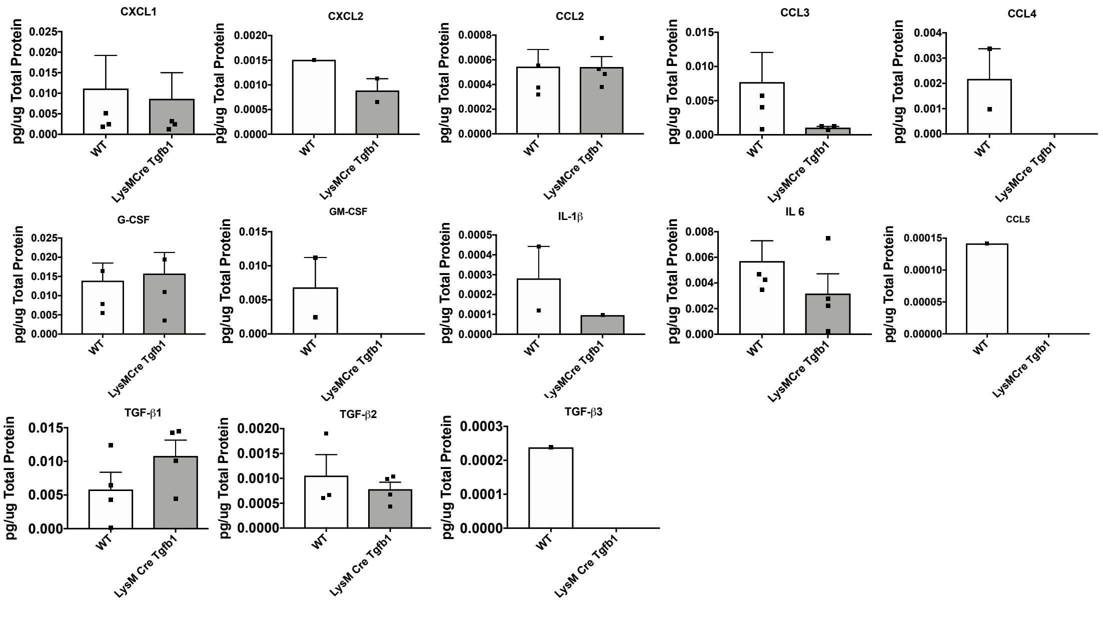

Supplemental Figure 6. Homogenate and Plasma levels of cytokines/chemokines after monocyte depletion using LysMCre-*Tgfb1*$^{fl/fl}$ mice (n=4 mice/group). Source data are provided as a Source Data file.

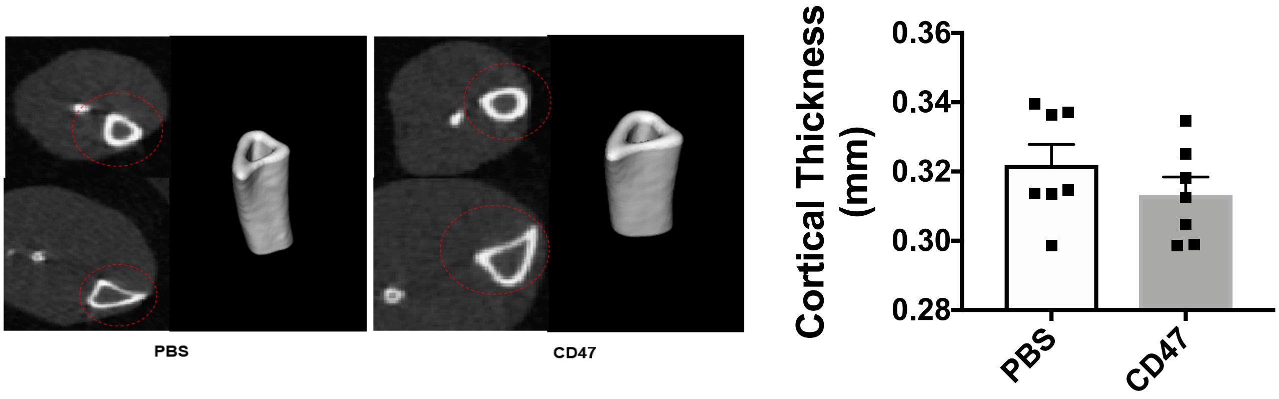

Supplemental Figure 7. Treatment with CD47 activating peptide p7N3 does not result in changes in tibial thickness of mice. Top: Quantification of mean cortical tibial thickness from MicroCT scans (n=7/group, t=1.103, df=12, p=0.292). Bottom: Representative MicroCT scan images from tibias of PBS and CD47 peptide (p7N3) treated mice. Source data are provided as a Source Data file.

Supplemental Table 1: Top gene expression of day 3 TGF-β expressing clusters.

p_val	avg_logFC	pct.1	pct.2	p_val_adj	cluster	gene
**0**	1.47036156	0.979	0.66	0	0	Plac8
**0**	1.32737263	0.907	0.353	0	0	Ifitm6
**3.73E-179**	1.31302633	0.817	0.512	6.57E-175	0	Chil3
**4.79E-299**	1.15385338	0.848	0.343	8.43E-295	0	Ly6c2
**1.97E-266**	1.07680069	0.98	0.844	3.47E-262	0	Thbs1
**0**	1.06899311	0.983	0.68	0	0	Hp
**0**	1.02867456	0.967	0.623	0	0	Gsr
**0**	0.98573785	0.825	0.316	0	0	Vcan
**2.27E-288**	0.94741447	0.832	0.315	3.99E-284	0	F10
**4.51E-283**	0.94567273	0.77	0.221	7.94E-279	0	Gm9733
**0**	2.4728712	0.84	0.292	0	1	Arg1
**5.01E-158**	2.05073848	0.97	0.884	8.81E-154	1	Spp1
**3.25E-66**	1.63075966	0.512	0.29	5.72E-62	1	Cxcl3
**5.61E-37**	1.12059119	0.165	0.048	9.87E-33	1	Mmp12
**5.00E-108**	1.06784211	0.988	0.947	8.81E-104	1	Pf4
**3.52E-133**	1.05879712	0.989	0.936	6.20E-129	1	Ctsl
**2.60E-160**	1.02772177	0.865	0.568	4.58E-156	1	Hilpda
**4.69E-17**	1.02137961	0.228	0.128	8.25E-13	1	Ppbp
**4.31E-81**	0.9504383	0.49	0.215	7.59E-77	1	Cd36
**3.46E-178**	0.9223303	0.988	0.899	6.10E-174	1	Mif
**1.72E-86**	0.77324378	0.933	0.832	3.03E-82	3	H2-Aa
**3.61E-111**	0.76874144	0.997	0.974	6.36E-107	3	Cd74
**3.40E-78**	0.72232396	0.906	0.748	5.99E-74	3	H2-Eb1
**1.79E-149**	0.67948462	0.991	0.816	3.14E-145	3	Aif1
**4.87E-164**	0.66808656	1	0.921	8.57E-160	3	Ctsc
**3.51E-87**	0.6639899	0.96	0.898	6.18E-83	3	H2-Ab1
**3.39E-86**	0.59492665	0.978	0.911	5.98E-82	3	Marcksl1
**4.67E-123**	0.57249586	0.974	0.758	8.22E-119	3	AF251705
**4.22E-79**	0.52282696	0.993	0.949	7.44E-75	3	Tgfbi
**1.81E-88**	0.51670001	0.979	0.716	3.19E-84	3	Ccr2

Supplemental Table 2: Common Macrophage Subsets at HO site in PBS and CD47 activating peptide treatment.

Cluster #		Gene
**PBS**	**CD47**	
4	1	Spp1
		Arg1
		Lgals3
		Cstb
		Cd36
		Prdx1
		Mmp12
3	3	Plac8
		Ccr2
		Cd52
		Il1b
		H2-Eb1
		H2-Aa
1	2	Sepp1
		C1qa
		Apoe
		C1qb
		C1qc
		Aif1
		Trem2
		Lyz2

Supplemental Table 3: Unique Macrophage Subsets at HO site with CD47 activating peptide treatment

Gene	Avg. Fold Change	% Expression in cluster	% Expression all other clusters	Adjusted p-val	Cluster #
**PBS**					
Ccl4	1.92741015	0.594	0.175	1.89E-171	9
Ccl3	1.428509938	0.544	0.125	4.42E-194	9
Mrc1	1.325374474	0.847	0.249	9.71E-263	9
Cd83	1.305317037	0.797	0.189	1.29E-302	9
Isg15	2.096509936	0.908	0.2	5.41E-177	12
Ccl12	1.981830311	0.638	0.093	6.41E-153	12
Ifit3	1.893926081	0.85	0.058	0	12
Irf7	1.803168506	0.932	0.187	1.11E-197	12
Fcgr1	1.502130958	0.937	0.243	6.60E-152	12
Ifit1	1.44619128	0.643	0.059	4.30E-247	12
**CD47**					
Folr2	1.688539551	0.671	0.099	0	6
Mrc1	1.623389766	0.925	0.243	0	6
Ccl12	1.615203405	0.58	0.077	0	6
Cbr2	1.444270202	0.629	0.071	0	6
F13a1	1.294819395	0.765	0.203	0	6
Fcrls	1.281069482	0.699	0.092	0	6
Clec10a	1.234401091	0.603	0.126	2.6929E-301	6
Cxcl3	4.773273925	0.963	0.1	4.6591E-167	12
Ccl3	4.597986709	1	0.22	3.71915E-95	12
Csf3	3.566634486	0.768	0.022	0	12
Serpinb2	3.413322798	0.707	0.011	0	12
Il1a	3.274139858	0.976	0.022	0	12
Arg1	2.644316173	0.939	0.114	1.8472E-131	12
Il1rn	2.623427277	0.915	0.218	7.91127E-72	12
Nos2	2.210727868	0.78	0.01	0	12
Inhba	2.065551886	0.805	0.063	1.3879E-168	12
Egln3	1.497285254	0.72	0.022	0	12
Ero1l	1.483284038	0.841	0.19	1.16011E-66	12

Supplemental Table 4: Taqman primer/probe assays used for QPCR.

Gene		Lot	Assay ID
Gapdh	FAM	1593842	Mm99999915_g1
Nos1	FAM	1732158	Mm01208059_m1
Arg1	FAM	1712842	Mm00475988_m1
Mrc1	FAM	1721427	Mm01329362_m1
Tgfβ1	FAM	1730281	Mm01178820_m1
