# Supplementary material for: Regulation of heterotopic ossification by monocytes in a mouse model of aberrant wound healing
Source: Nat Commun. 2020 Feb 5;11:722. doi: 10.1038/s41467-019-14172-4 (PMC7002453; doi:10.1038/s41467-019-14172-4)
Supplement: Supplementary file 3 — Reporting Summary [file 41467_2019_14172_MOESM3_ESM.pdf]

nature research

Corresponding author(s): Dr. Benjamin Levi, MD
1150 W. Medical Center Drive
MSRBII Room A574
Ann Arbor, MI, 48109
blevi@umich.edu

Last updated by author(s): 12/11/2019

Reporting Summary

Nature Research wishes to improve the reproducibility of the work that we publish. This form provides structure for consistency and transparency in reporting. For further information on Nature Research policies, see Authors & Referees and the Editorial Policy Checklist .

Statistics

For all statistical analyses, confirm that the following items are present in the figure legend, table legend, main text, or Methods section.

n/a	Confirmed	
☐	☒	The exact sample size (n) for each experimental group/condition, given as a discrete number and unit of measurement
☐	☒	A statement on whether measurements were taken from distinct samples or whether the same sample was measured repeatedly
☐	☒	The statistical test(s) used AND whether they are one- or two-sided<br>*Only common tests should be described solely by name; describe more complex techniques in the Methods section.*
☒	☐	A description of all covariates tested
☐	☒	A description of any assumptions or corrections, such as tests of normality and adjustment for multiple comparisons
☐	☒	A full description of the statistical parameters including central tendency (e.g. means) or other basic estimates (e.g. regression coefficient) AND variation (e.g. standard deviation) or associated estimates of uncertainty (e.g. confidence intervals)
☐	☒	For null hypothesis testing, the test statistic (e.g. F, t, r) with confidence intervals, effect sizes, degrees of freedom and P value noted<br>*Give P values as exact values whenever suitable.*
☒	☐	For Bayesian analysis, information on the choice of priors and Markov chain Monte Carlo settings
☒	☐	For hierarchical and complex designs, identification of the appropriate level for tests and full reporting of outcomes
☒	☐	Estimates of effect sizes (e.g. Cohen's d, Pearson's r), indicating how they were calculated

Our web collection on statistics for biologists contains articles on many of the points above.

Software and code

Policy information about availability of computer code

Data collection	10x genomics; BD FACSDIVA (v8.0), Leica TCS SP8 and Leica SP5 Confocal Scanning Microscope, ABI 7900HT, bioplex 200, Bruker MicroCT,IVIS spectral imager, Olympus BX-51
Data analysis	SPSS (v25), FlowJo (v10.5.0), LAS X, GE systems Parallax Microview, ImageJ (e1.52), Prism (v.7),cell ranger, R studio, GSEA, Olympus DP Viewer

For manuscripts utilizing custom algorithms or software that are central to the research but not yet described in published literature, software must be made available to editors/reviewers. We strongly encourage code deposition in a community repository (e.g. GitHub). See the Nature Research guidelines for submitting code & software for further information.

Data

Policy information about availability of data

All manuscripts must include a data availability statement. This statement should provide the following information, where applicable:

- Accession codes, unique identifiers, or web links for publicly available datasets
- A list of figures that have associated raw data
- A description of any restrictions on data availability

Single cell data is deposited in GEO (GSE126060). RNA sequencing data of human data was obtained from GEO and is available online (GSE37069). Mouse RNA sequencing is deposited in GEO (GSE126118).

Field-specific reporting

Please select the one below that is the best fit for your research. If you are not sure, read the appropriate sections before making your selection.

- [x] Life sciences
- [ ] Behavioural & social sciences
- [ ] Ecological, evolutionary & environmental sciences

For a reference copy of the document with all sections, see nature.com/documents/nr-reporting-summary-flat.pdf

Life sciences study design

All studies must disclose on these points even when the disclosure is negative.

Sample size	No power calculation was performed. Sample size was determined during experimental design based on the experiment and type of measurements being performed based on previous experience with data variation with each technique.
Data exclusions	No data exclusion was performed.
Replication	Single cell experiments were performed in triplicate for each time point to determine mouse variability. Protein luminex were performed once with at least three mice in each group. Histology samples were performed with at least 3 mice for each treatment/group. Mouse treatment studies (clodronate/p7N3) were performed in at least triplicate.
Randomization	All mice for this study were randomized. Litter mates were used in experiments where possible.
Blinding	Blinding in this study was accomplished by having one investigator performing the surgeries and a second independent investigator collecting and analyzing the data. Once data was analyzed, treatment groups or mouse genotypes were revealed.

Reporting for specific materials, systems and methods

We require information from authors about some types of materials, experimental systems and methods used in many studies. Here, indicate whether each material, system or method listed is relevant to your study. If you are not sure if a list item applies to your research, read the appropriate section before selecting a response.

Materials & experimental systems

n/a	Involved in the study	
[ ]	[x]	Antibodies
[x]	[ ]	Eukaryotic cell lines
[x]	[ ]	Palaeontology
[ ]	[x]	Animals and other organisms
[x]	[ ]	Human research participants
[x]	[ ]	Clinical data

Methods

n/a	Involved in the study	
[x]	[ ]	ChIP-seq
[ ]	[x]	Flow cytometry
[x]	[ ]	MRI-based neuroimaging

Antibodies

Antibodies used

FITC:Ly6C (BD bioscience) 1:200 Clone:AL21
BV510:CD11b (BD bioscience) 1:100 Clone: M1/70
APCH7:Ly6G (BD bioscience) 1:100 Clone: 1A8
BB700:F4/80 (BD bioscience) 1:100 Clone: T45-2342
BV421:CD206 (BD bioscience) 1:100 Clone: C068C2
rat anti-PDGFRa (santa cruz sc-338) 1:50 Clone: C-20
rabbit anti-sox9 (abcam ab185230) 1:50 Clone: EPR14335
rabbit anti-TGFB1 (Novus NBP1-45891) 1:50 Clone: N/A
rat anti-CD16/32 (BD 553141) 1:50 Clone: 2.4G2
goat anti-CD206 (RD AF2535) 1:50 Clone: N/A
rat anti-F4/80 (abcam ab6640) 1:50 Clone: A3-1
rabbit anti-pSMAD3 (Novus NBP1-77836) 1:50 Clone: N/A
donkey anti-rat alexa fluor 594 (Jackson 712-586-153) 1:200
donkey anti-rabbit alexa fluor 488 (Invitrogen A-21208) 1:200
rabbit anti-human CD68 (Cat No. ab125212, Abcam) 1:100
Biotinylated secondary antibody (Cat No. BA-1000, Vector Laboratories) 1:200
mouse anti-human TGF-β1 antibody (Cat No. MAB240, R&D Systems) 1:50
IMPRESS-AP reagent Anti-Mouse IgG (Cat No. MP-5402, Vector Laboratories)
anti-mouse SIRPa (Catalog #BE0322, Bioxcell) 100ug/mouse

	Isotype control rIgG1k (Catalog# BE0088, Bioxcell) 100ug/mouse
Validation	Compensation of samples was done with antibody labeled beads, and isotype controls were used to determine negative populations by flow cytometry. Western antibodies were validated in house and all antibodies used had validated statement provided on the product manufacturer websites for use with proper applications.

Animals and other organisms

Policy information about studies involving animals; ARRIVE guidelines recommended for reporting animal research

Laboratory animals	Mice: young adult male (6–10 weeks old) C57BL/6J mice were purchased from Jackson Laboratories (Bar Harbor, ME). C57BL/6J background LysM-Cre/Tgfb1fl/fl (compared to strain and age matched control), LysM-Cre/iDTRfl/fl (compared to littermate control) and LysM-Cre/mTmGfl/fl were bred by crossing the respective alleles and then crossing heterozygous mice for both traits to obtain mice undergoing experiments. Litter mates were used as controls where possible.
Wild animals	No wild animals were used.
Field-collected samples	N/A
Ethics oversight	All animal procedures were carried out in accordance with the guidelines provided in the Guide for the Use and Care of Laboratory Animals from the Institute for Laboratory Animal Research (ILAR, 2011) and were approved by the Institutional Animal Care and Use Committee (IACUC) of the University of Michigan (PRO0007390)

Note that full information on the approval of the study protocol must also be provided in the manuscript.

Flow Cytometry

Plots

Confirm that:

- [x] The axis labels state the marker and fluorochrome used (e.g. CD4-FITC).
- [x] The axis scales are clearly visible. Include numbers along axes only for bottom left plot of group (a 'group' is an analysis of identical markers).
- [x] All plots are contour plots with outliers or pseudocolor plots.
- [x] A numerical value for number of cells or percentage (with statistics) is provided.

Methodology

Sample preparation	Soft tissue around the injury site was dissected from the posterior compartment between the muscular origin and calcaneal insertion of Achilles tendon at the indicated time points. Corresponding soft tissue including intact Achilles' tendon was also harvested from the uninjured contralateral hind limb. Tissue was digested for 45 minutes in 0.3% Type 1 Collagenase and 0.4% Dispase II (Gibco) in Roswell Park Memorial Institute (RPMI) medium at 37°C under constant agitation at 120rpm. Digestions were subsequently quenched with 10% FBS RPMI and filtered through 40µm sterile strainers. Specimens were blocked with anti-mouse CD16/32 and subsequently stained using the following antibodies: FITC:Ly6C, BV510:CD11b, APCH7:Ly6G, BB700:F4/80 (BD), BV421:CD206 and BV650:MHCII (BioLegend).
Instrument	LSR Fortessa, BD Aria III
Software	BD FacsDiva (v8.0) Flow Jo (v10)
Cell population abundance	Cellular composition of the B/T site had previously not been defined, we determined that Neutrophils represented 19% of total cells at day 2, Monocytes represented about 40% of all cell at day 2 and 15% of all cells 3 weeks after Burn Tenotomy injury. This was determined by flow cytometry and is shown in our data in Figure 2d.
Gating strategy	Our gating strategy is represented in figure 2c, and is as follows: live cells were gated based on SSC-A and FSC-A. Doublet exclusion was performed using SSC-H/SSC-W gating. Viability, determined by PI, gated on live cells, and then CD11b by Ly6G determined Neutrophils and monocytes, and these were further separated by Ly6C expression.

- [ ] Tick this box to confirm that a figure exemplifying the gating strategy is provided in the Supplementary Information.
